# Targeting cytotoxic lymphocyte antigen 4 (CTLA-4) in breast cancer

**DOI:** 10.1186/s40001-024-01901-9

**Published:** 2024-07-02

**Authors:** Maryam Jama, Yasser Tabana, Khaled H. Barakat

**Affiliations:** 1 Faculty of Pharmacy and Pharmaceutical Sciences, University of Alberta, Edmonton, Canada; 2 Li Ka Shing Institute of Virology, University of Alberta, Edmonton, Canada; 3 Faculty of Medicine and Dentistry, University of Alberta, Edmonton, Canada

## Abstract

Breast cancer (BC) has a high mortality rate and is one of the most common malignancies in the world. Initially, BC was considered non-immunogenic, but a paradigm shift occurred with the discovery of tumor-infiltrating lymphocytes (TILs) and regulatory T cells (Tregs) in the BC tumor microenvironment. CTLA-4 (Cytotoxic T-lymphocyte-associated protein 4) immunotherapy has emerged as a treatment option for BC, but it has limitations, including suboptimal antitumor effects and toxicity. Research has demonstrated that anti-CTLA-4 combination therapies, such as Treg depletion, cancer vaccines, and modulation of the gut microbiome, are significantly more effective than CTLA-4 monoclonal antibody (mAB) monotherapy. Second-generation CTLA-4 antibodies are currently being developed to mitigate immune-related adverse events (irAEs) and augment antitumor efficacy. This review examines anti-CTLA-4 mAB in BC, both as monotherapy and in combination with other treatments, and sheds light on ongoing clinical trials, novel CTLA-4 therapeutic strategies, and potential utility of biomarkers in BC.

**Graphical Abstract:**

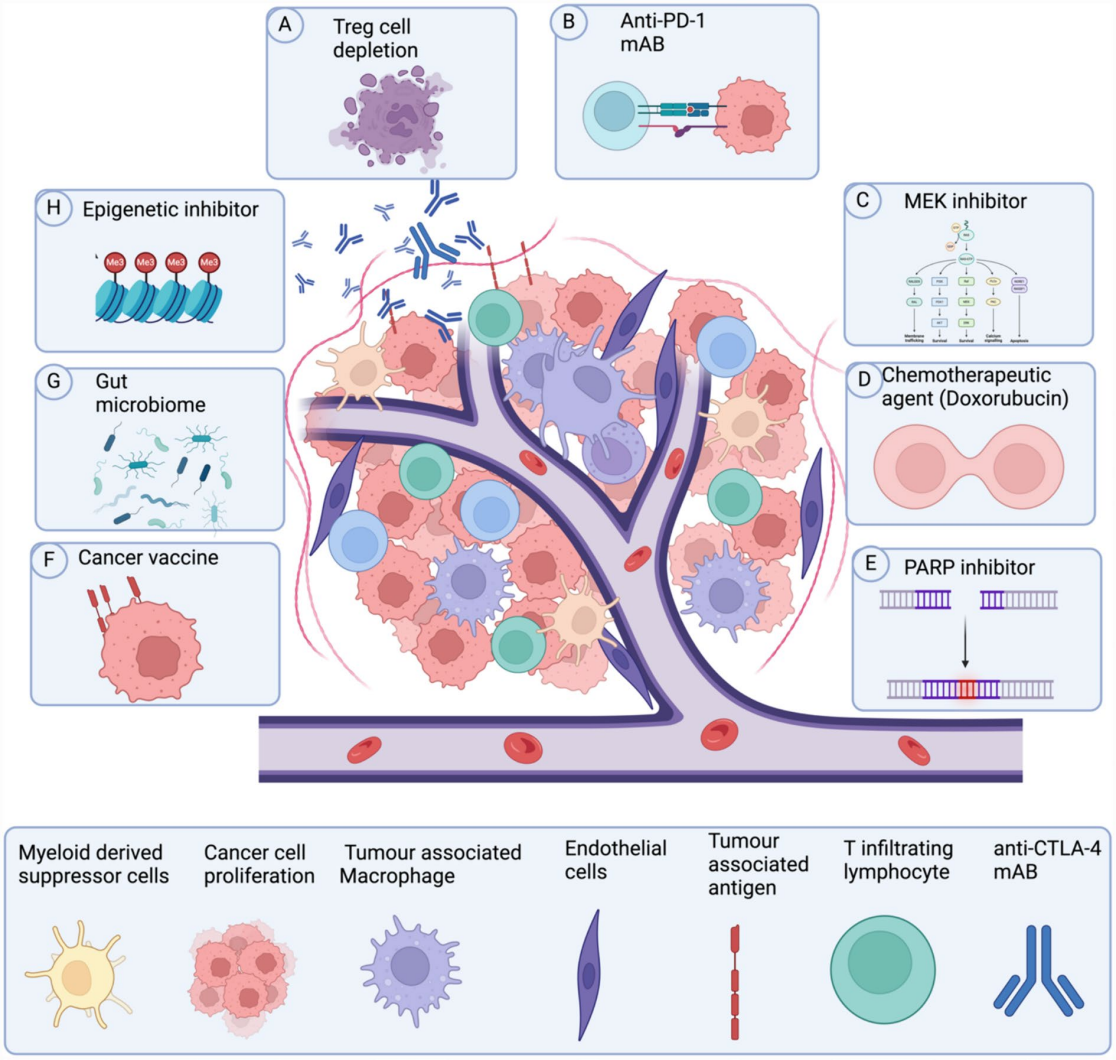

## Introduction

Breast cancer, which is one of the most prevalent malignancies and has the fifth-highest mortality rate among all cancers, has traditionally not been considered immunogenic [67, 96]. However, recent studies have indicated the presence of tumor-infiltrating lymphocytes (TIL) and regulatory T (Treg) cells in breast cancer, providing the rationale for immunotherapy as a potential treatment option, particularly for triple-negative breast cancer (TNBC), which currently lacks targeted treatment and is not responsive to standard therapies for a large proportion of patients [45, 105]. Immunotherapy is currently under investigation in the context of breast cancer and the identification of potential biomarkers that may predict the response to treatment.

**Fig. 1:**
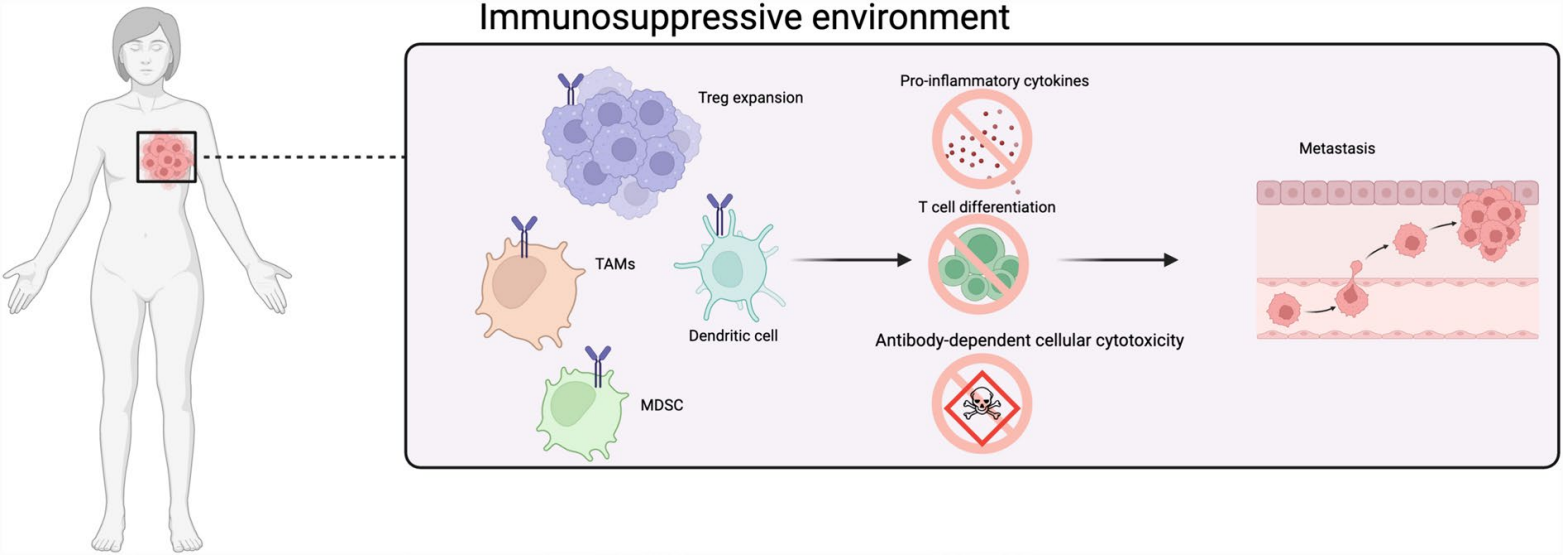
Extrinsic therapeutic targets for CTLA-4 therapy in breast cancer. CTLA-4 overexpression reduces pro-inflammatory cytokines, T-cell effector differentiation, and antibody-dependent cellular cytotoxicity, resulting in tumor proliferation and metastases of breast cancer

Tumors co-opt immune checkpoint proteins such as CTLA-4 to create an immunosuppressive tumor microenvironment (TME), circumvent immune surveillance, and promote tumor progression (Fig. 1) [36]. Notably, CTLA-4 is found in T cells, non-lymphoid cells, B cells, dendritic cells, stromal cells, and tumors [60, 72]. CTLA-4 negatively regulates T effector cell function by outcompeting CD28/CD80 co-stimulatory receptors and binding to their shared ligand, B7-1, thereby inhibiting T-cell activation with higher avidity and affinity [16]. CTLA-4 bound to B7-1 weakens the co-stimulatory signal, producing overall immunosuppressive activity [89]. Furthermore, CTLA-4 interferes with the co-stimulatory signal by removing B7-1 ligands from the surface of antigen-presenting cells via trans-endocytosis and trogocytosis (APC). CTLA-4 removal from the surface of APCs reduces T effector cell function and proliferation [83]. Moreover, CTLA-4 indirectly modulates immunosuppression in the tumor microenvironment (TME) by limiting CD4 + T cells' clonal expansion, which is essential for targeting malignant tumor cells through direct killing or enhancing cytotoxic T-cells and B-cells immune response [6, 46, 69, 102]. CD4 + T-cell reduction diminishes pro-inflammatory cytokine levels, including interleukin 2 (IL-2) and tumor necrosis factor α (TNF-α) as observed in many tumors, including BC [37, 43, 72, 98, 123]. Consequently, CTLA-4 dampens T-cell responses by interacting with a network of immune and tumor cells, producing an immunosuppressive environment. Hence, CTLA-4 humanized monoclonal antibodies (mAB) such as ipilimumab and tremelimumab have demonstrated great clinical benefits in many different malignancies, including melanoma, lung carcinoma, and renal cell carcinoma, but not in BC [20, 36, 116]. CTLA-4 mAB’s has suboptimal antitumor activity and high toxicity effect of anti-CTLA-4 monotherapy in BC; thus, anti-CTLA-4 combination therapy is being explored as an alternative for breast cancer [57]. This review will focus on the mechanism of action of anti-CTLA-4 mAB alone and in combination with other therapies in BC, current clinical trials with anti-CTLA-4 in BC patients, and novel therapeutic approaches to enhance the efficacy and minimize the toxicity of anti-CTLA-4 in BC patients.

CTLA-4 blockade depends on several factors including Treg cells, T-cell infiltration, CD8 + T-cell activation, and tumor-associated macrophage (TAMs) recruitment (Fig. 1). Treg cells are a subpopulation of immune cells required for self-tolerance and to prevent autoimmune diseases [102]. They constitutively express CTLA-4, which inhibits CD4 + and CD8 + cytotoxic T cells in the TME [25, 55, 79]. For instance, Treg cell depletion regressed tumors within a month and increased survival by > 80 days in murine models [74, 95]. Similarly, other studies have demonstrated that Treg cell depletion in mice promoted lymphocyte recruitment and decreased tumor growth rate, implicating Treg's role in tumor promotion [17, 39]. Furthermore, a relatively high level of Treg cells in the TME is associated with poor prognosis in various cancer types, including breast cancer [21]. Numerous studies have demonstrated that CTLA-4 therapies deplete tumor resident Treg cells and downregulate cytotoxic T-cell activity [44, 51, 70, 77, 79, 82, 90, 93, 103, 109]. Treg cells mediate immunosuppression through various mechanisms, such as IL-2 depletion and immunosuppressive cytokine production. Notably, the primary mechanism of action of CTLA-4 mAB is antibody-dependent cellular cytotoxicity (ADCC) of Treg cells (Fig. 1) [8, 11, 13, 18, 49, 55, 94, 97, 108]. The efficient antitumor effects of CTLA-4 blockade depend on the ADCC of Treg cells, as CTLA-4 blockade alone without ADCC has insufficient antitumor activity [115]. Another study revealed that ipilimumab has better activity in patients with FcγR variants that enhance ADCC activity, further implicating the importance of ADCC activity in achieving a robust response [111]. Although anti-CTLA-4 mAB effectively promotes tumor regression in some malignancies, it is insufficient in BC [20, 36, 110]. Nevertheless, preclinical studies have shown that anti-CTLA-4 combination therapy can effectively control tumor growth in BC. Taylor et al. [110] demonstrated CTLA-4 and PD-1 blockade, combined with adoptive cell therapy, significantly delayed tumor growth and prolonged survival by stringently depleting Treg cells in TNBC murine models. Conversely, CTLA-4 and PD-1 blockade alone are insufficient to suppress tumor growth [110]. These findings suggest that anti-CTLA-4 combination therapy could effectively suppress aggressive breast tumor growth compared with anti-CTLA-4 alone. Furthermore, efforts are underway to create novel anti-CTLA-4 mABs with a modified fragment crystallizable (FC) region, promoting stringent Treg cell depletion and robust antitumor activity alone. Gan et al. [28] developed a novel anti-CTLA-4 heavy chain antibody (HCAb 4003-1) engineered to enhance Treg cells' depletion. These findings demonstrate HCAb 4003-1 had higher antitumor activity and a shorter serum half-life than ipilimumab, implicating its minimal toxicity effect [28]. Similarly, developed CTLA-4 mABs with a modified FC region, which demonstrated a robust antitumor response associated with ADCC depletion of Treg cells. These antibodies are promising candidates for future generation of anti-CTLA-4 therapies. A significant concern regarding anti-CTLA-4 mAB is its adverse toxic effects [26]. For instance, ipilimumab lysosomally degrades ~ 50% of CTLA-4, causing severe immunotherapy-related adverse effects (irAEs). However, pH-sensitive CTLA-4 antibodies have been developed (HL12 and HL32) that circumvent immune-related adverse events (irAEs) while producing antitumor effects [122]. The pH-sensitive antibodies bound to CTLA-4 dissociate after lysosomal endocytosis and are recycled back to the cell surface, minimizing CTLA-4 degradation and the overall iRAE. In contrast, conventional mAB is bound to CTLA-4 and is subsequently engulfed by lysosomes and degraded, reducing CTLA-4 levels and inducing iRAE [122]. Besides reducing toxicity levels, pH-sensitive antibodies have high bioavailability, enhancing intratumoral Treg cell depletion and ADCC targeting of CTLA-4, thus amplifying their effectiveness [122]. These findings suggest a novel approach for the development of second-generation antibodies targeting CTLA-4 with high potency and minimal toxicity [122].

## Anti-CTLA-4 mAB combination therapy in BC

Several studies have shown that targeting multiple immune inhibitory pathways through combination therapy improves anti-CTLA-4 efficacy through several mechanisms [23, 92]. First, it prevents the compensatory upregulation of other inhibitory checkpoint pathways [19]. Second, immune cells are targeted systematically rather than locally through their distinct and complementary roles (i.e., in the lymph node and tumor site) (Fig. 1) [78]. Third, various immune-infiltrating cells (Tregs and TAMS) are targeted, inducing a stronger inflammatory response, which has implications for immunotherapy resistance (Fig. 1) [92]. For instance, studies have shown that targeting both CTLA-4 and PD-1 expands cytotoxic T cells and transforms tumor-associated monocytes and macrophages to a pro-inflammatory (M1) phenotype through increased IFN-γ secretion, producing an inflammatory tumor microenvironment (TME) [5, 19, 62]. Furthermore, a preclinical study demonstrated that CTLA-4 and PD-1 ICI increased CD8 + T-cell intratumoral infiltration compared to anti-CTLA-4 monotherapy, increasing the rate of tumor-free survival in melanoma mouse models by ~ 65% [19]. Overall, the combination of CTLA-4 and PD-1 immune checkpoint inhibitors boosts antitumor efficacy by influencing intratumoral lymphocyte and myeloid cell components [35, 84, 118]. A single-arm clinical study analyzed the efficacy of tremelimumab in combination with durvalumab (a PD-1 inhibitor) in 18 metastatic breast cancer patients with MBC. The cohort had an objective response rate (ORR) of 17% and 0% in the ER + patients and 43% in the TNBC group. However, the benefits of anti-CTLA-4 and anti-PD-1 combination therapy did not outweigh the risks, and the study was terminated in the second phase (Santa-Maria et al. 2017). Another study investigated ipilimumab in combination with nivolumab in advanced chemotherapy-refractory metaplastic breast cancer. Although the study reached its primary endpoint, 65% of patients experienced significant irAEs [2]. By lowering the dosage of ipilimumab or using newer anti-CTLA-4 formulations irAE could be reduced in patients [4, 114].

Although the combination of CTLA-4 and PD-1 mABs proved to be efficacious, it is inadequate for high levels of intratumoral macrophages in advanced stages of cancer [118]. Neoadjuvant anti-CTLA-4 therapy has been reported to provide a robust antitumor immune response compared to ICI alone in aggressive tumors [87]. An ongoing clinical study is investigating neoadjuvant anti-CTLA-4 therapy in TNBC patients (NCT03546686). This study investigated the safety of administering CTLA-4 and PD-1 immunotherapy in combination with cryoablation or standard care in patients with HER2-negative advanced breast cancer following neoadjuvant chemotherapy. Preclinical findings revealed that this treatment induced a robust immunological response against the tumor both locally and systemically [22, 50] (Table 1).

**Table 1:** Clinical trial testing Anti-CTLA-4 combination therapy in breast cancer patients

Immune intervention	Phase	Participants	Trial subject	Status/Results	Clinical trial
Ipilimumab + nivolumab	II	30	Hypermutated HER2-negative breast cancer	Ongoing	NCT03546686
Ipilimumab, nivolumab, cryoablation	II	160	HER2-negative advanced breast cancer after neoadjuvant chemotherapy	Ongoing	NCT03546686
Ipilimumab, nivolumab, pegylated liposomal doxorubicin, and cyclophosphamide	IIb	75	Breast cancer	Ongoing	NCT02069158
Brain irradiation and Tremelimumab In metastatic breast cancer	N/A	17	Metastatic breast cancer	Completed	NCT02563925

**Fig. 2:**
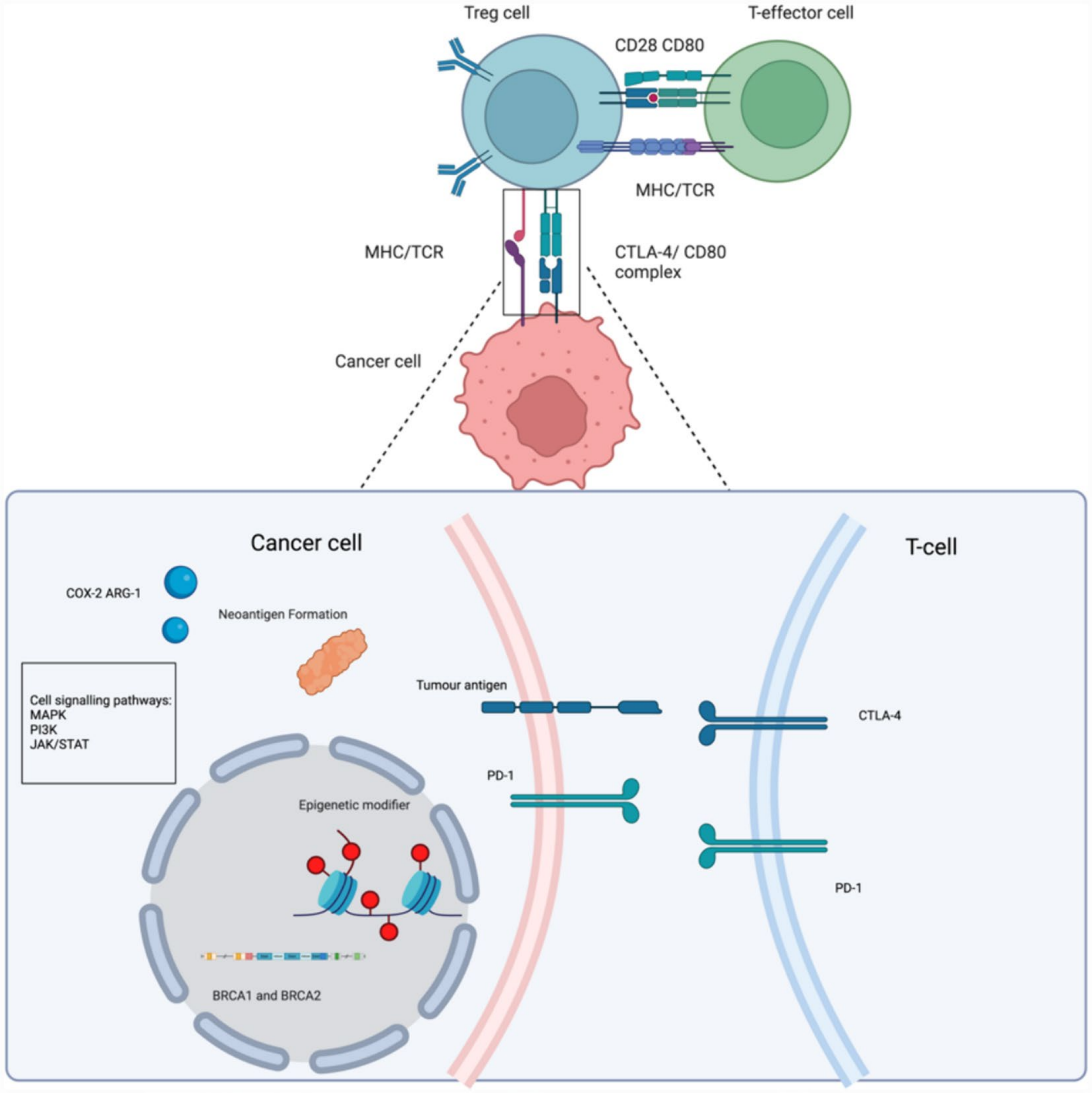
Intrinsic therapeutic targets for CTLA-4 combination therapy include tumor antigens, PD-1 receptor, epigenetic modifiers, BRCA1 and BRCA2, and immunosuppressive mediators (COX-2 and ARG-1), in addition to MAPK and PI3K signaling pathways in cancer cells

Anti-CTLA-4 mAB in combination with targeted therapy such as chemotherapy, epigenetic modulators, poly (ADP-ribose) polymerase (PARP) inhibitors, and radiotherapy enhances its therapeutic efficacy compared to monotherapy (Fig. 2) [76]. For example, anti-CTLA-4 mAB, in combination with MAPK inhibitors in TNBC, had a stronger antitumor immunity than anti-CTLA-4 mAB alone (Fig. 2). [33] reported that anti-CTLA-4 mAB in combination with Selumetinib, a MEK1/2 small-molecule inhibitor (SMI), abolished the upregulation of immunosuppressive mediators (Cox-2 and Arg1-), whereas anti-CTLA-4 monotherapy increased their expression in TNBC mouse models (Fig. 2) [33]. Similarly, another study reported that anti-CTLA-4 mAB in combination with PI3Kα inhibitor provided synergistic antitumor activity, regressed breast tumors by 60%, and sensitized the tumor to ICI compared to ICI alone (Fig. 2) [119]. These findings provide a rationale for multiple ongoing clinical studies investigating the efficacy and safety of gedatolisib (PI3Kα inhibitor) in combination with anti-CTLA-4 mAB in patients with metastatic BC [119]. A clinical trial is currently underway to examine the impact of combining anti-CTLA-4 mAB with paclitaxel and gedatolisib on both antitumor efficacy and tolerance in breast cancer patients. (Table 1). Another clinical study investigated the effects of tremelimumab in combination with brain radiotherapy and trastuzumab (a HER2 inhibitor) in patients with ER + metastases (NCT02563925) [85]. Furthermore, chemotherapeutic agents such as paclitaxel, doxorubicin, and gemcitabine prime the immune system by suppressing myeloid derived suppressor cells (MDSCs) enhancing the efficacy of anti-CTLA-4 blockade [7, 27 ]. Hence, a phase IIb clinical study assessed liposomal doxorubicin and cyclophosphamide alone and in combination with ipilimumab and nivolumab in patients with BC (NCT03409198) (Table 1). Another example of CTLA-4 combination treatment enhancing efficacy pertains to targeting mutations in Breast Cancer genes1/2 (BRCA1/2), which are genes involved in DNA repair (Fig. 2) [84]. Notably, high levels of T-cell recruitment and immune gene expression characterize BRCA-mutated tumors and correlate with patients' cytotoxic responses to ICI [73]. Studies have demonstrated that breast tumors with BRCA mutations treated with cisplatin elicited an effective antitumor immune response through a high mutational burden [14, 66, 101, 117]. Similarly, combining CTLA-4 mAB, PD1 mAB, and cisplatin in BRCA-deficient breast cancer mouse models resulted in synergistic cytotoxicity. This combination treatment diminished FOXP3 + Treg cell levels and elevated CD4 + and CD8 + T-cell levels (*p* < 0.05), significantly reducing tumor growth [73]. Similarly, a preclinical study demonstrated that treating BRCA-deficient immunocompetent ovarian cancer models with ipilimumab and veliparib (PARP inhibitor) enhanced IFN-gamma production and T-cell infiltration [38]. Ipilimumab and veliparib led to synergistic antitumor activity, increasing long-term survival in murine models [38]. Furthermore, a phase 1 clinical trial is underway to examine the effects of combining CTLA-4 mAB with PARP inhibitors in patients diagnosed with BRCA-deficient ovarian cancer. The initial findings from this study indicated positive therapeutic outcomes coupled with favorable tolerability (NCT02571725) (Table 1) [3]. Thus, investigating CTLA-4 mAB in combination with PARP inhibitors in patients with BRCA-mutated BC would be promising. In summary, these findings indicate that anti-CTLA-4 personalized therapy has great potential in BC.

## Novel immunotherapeutic strategies for targeting CTLA-4

### Anti-CTLA-4 and cancer vaccines

Cancer vaccines are an appealing anti-CTLA-4 therapeutic strategy because they enhance immunogenic response and tumor specificity by targeting neoantigens [90]. A previous study demonstrated that combining anti-CTLA-4 mAB and targeting mucin1 (MUC1), a tumor antigen, with cancer vaccines led to synergistic activation and cytotoxic T-cell expansion [56, 65, 71] (Fig. 2). Similarly, an in vivo study demonstrated that anti-CTLA-4 and anti-PD-1 mABs combined with an oncolytic virus (GM-CSF) increased CD8 + T cells and memory T cells and decreased Treg cell levels in TNBC orthotropic immune-competent murine models. Moreover, this combination therapy increased T helper type 1 cell (TH1) inflammatory cytokines, induced tumor cell apoptosis, reduced tumor growth by ~ 50%, and prolonged survival in TNBC murine models [121]. Another study demonstrated that anti-CTLA-4 combined with DNA vaccines also had synergistic effects, resulting in significant tumor regression compared to anti-CTLA-4 monotherapy [90]. Furthermore, clinical trials are testing tremelimumab as an adjuvant for breast cancer vaccines to enhance TIL recruitment and induce potent cytotoxic responses [30] (NCT02643303). The combination of anti-CTLA-4 therapy with cancer vaccines presents therapeutic promise, particularly for breast cancer patients with poor immunogenicity.

### Anti-CTLA-4 and microbiome

The efficacy of anti-CTLA-4 therapy also depends on commensal bacteria composition as studies have shown the gut microbiome is involved in breast cancer development and response to therapy [40, 52, 80, 99, 112]. A recent study reported that CTLA-4 blockade, in combination with introducing strains such as Bacteroides fragilis, germ-free, or antibiotics, improved the efficacy of anti-CTLA-4 therapy by further polarizing the TH1 T helper cells [68]. Furthermore, oral administration of probiotics such as Bacteroides fragilis and Burkholderia cepacia improved the irAE toxicity of anti-CTLA-4 therapy [88]. An ongoing clinical trial is examining the gut microbiota in response to ICI in BC patients (NCT02752685) [53]. These preliminary results indicate that gut microbial bacteria influence anti-CTLA-4 efficacy and antitumor response in breast cancer, making the gut microbiome therapeutically relevant for immunotherapy.

### Anti-CTLA-4 and epigenetic modulation

CTLA-4 mAB in combination with epigenetic modifiers is another promising therapeutic strategy for enhancing anti-CTLA-4 efficacy [61] (Fig. 2). The expression of CTLA-4, PD-1, and PD-L1 is enhanced by DNA hypomethylating agents [63]. Consistent with this study, Enhancer of Zeste 2 Polycomb Repressive Complex 2 subunit (EZH2), a methyltransferase enzyme, has been reported to be crucial for differentiating and maintaining Treg cells [120]. Notably, anti-CTLA-4 mABs have been reported to increase EZH2 expression in the peripheral T cells of treated patients. EZH2 inhibition in combination with anti-CTLA-4 mAB enhanced the antitumor immune response in murine models, as evidenced by the increased ratio of Treg to T effector cells and enhanced cytotoxicity of T effector cells compared to anti-CTLA-4 monotherapy [31]. Another study reported that histone deacetylase (HDAC) inhibitors combined with anti-CTLA-4 mAB and/or PD-1 mAB downregulated MDSC in murine mammary models, thereby increasing antitumor immunity [47]. Similarly, ICIs with histone deacetylases or DNA methyltransferases treat more than 80% of metastatic TNBC tumors, with MDSCs as the primary target [47]. Furthermore, the combination of anti-CTLA-4 mAB and HDAC inhibitors enhanced CD4 + T-cell infiltration and displayed synergistic antitumor activity [47]. These preclinical observations provide evidence for the rationale of combining epigenetic modulators with anti-CTLA-4 therapy in breast cancer [15, 47, 48, 75] (Table 1).

## Future directions for anti-CTLA-4 treatments

### Small-molecule CTLA-4: B7-1 inhibitors

While antibodies directed against CTLA-4, such as ipilimumab, have shown considerable effectiveness, small-molecule inhibitors (SMIs) are more advantageous because of their permeability, lower production costs, prolonged half-lives, and organ-specific targeting [100, 107]. CTLA-4 is currently deemed undruggable, with no known binding pocket at the ligand-binding interface [102]. These peptides and allosteric sites for CTLA-4 have yet to be determined through computational techniques such as molecular dynamic simulations [86, 104]. Nevertheless, some SMIs target CTLA-4 indirectly by blocking B7-1 and its interaction with CTLA-4 and CD28 [100] (Table 3). For instance, (8) and (9) target [24, 32]. Huxley et al. [41] also describe small-molecule inhibitors targeting B7-1 with high specificity and low nanomolar affinity [41]. Similarly, these small molecules were reported to antagonize the CTLA-4 interaction by occluding its binding site. Nonetheless, a limitation of these B7-1 SMIs is their capacity to hinder IL-2 secretion, which is necessary for pro-inflammation [41]. Currently, no CTLA-4 SMI is available on the market [100]. The trajectory of CTLA-4 therapy should center on developing SMIs that directly target CTLA-4 or epigenetically, as these agents offer more significant advantages than monoclonal antibodies.

### Anti-CTLA-4 therapy biomarkers

**Fig. 3:**
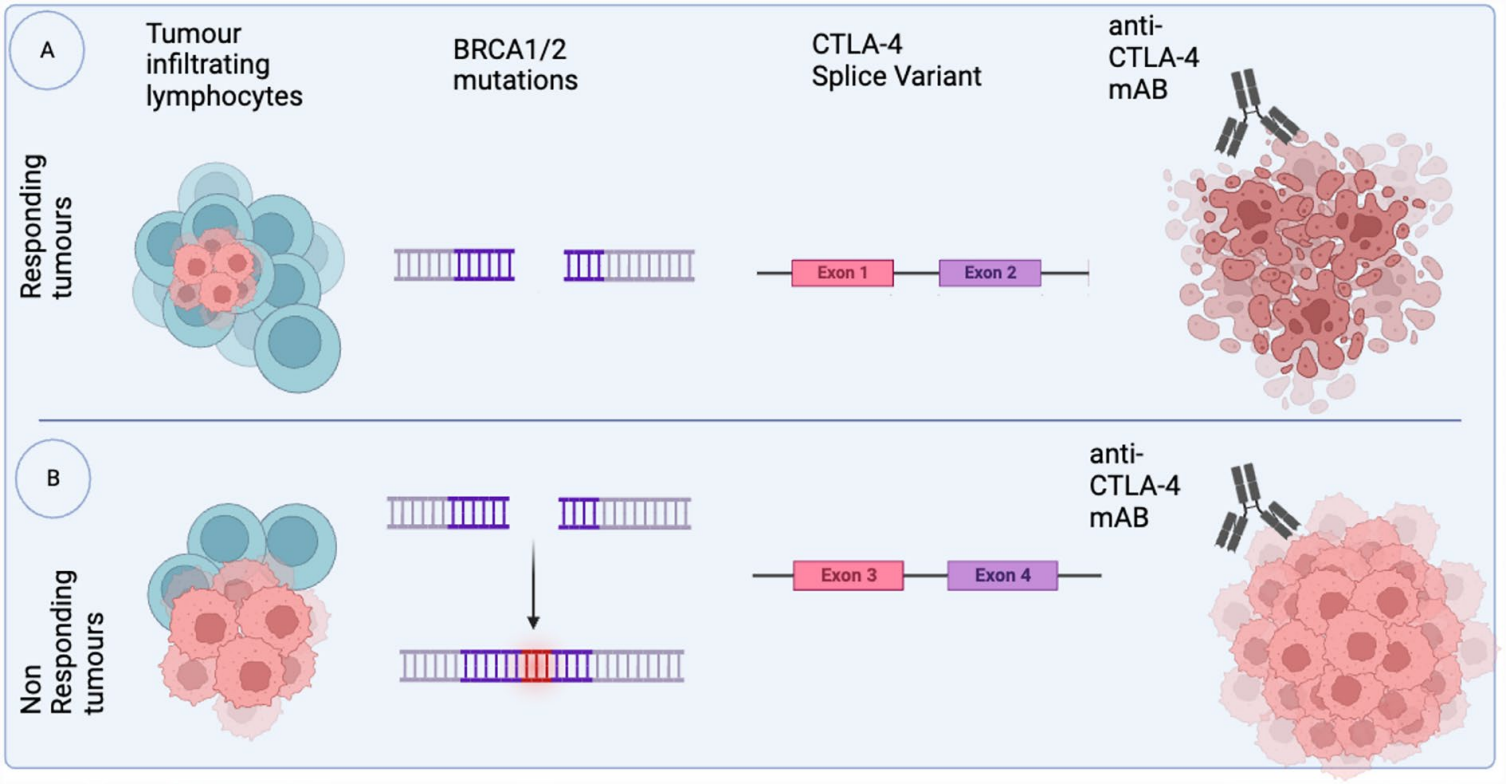
Breast cancer anti-CTLA-4 potential biomarkers; BRCA1/2, CTLA-4 SNPs, tumor-infiltrating lymphocytes

One of the biggest pitfalls of the CTLA-4 mAB is its ability to predict which patients will respond to and benefit from treatment (Fig. 3). Patients could be stratified into responsive and non-responsive groups by assessing predictive biomarkers such as single nucleotide polymorphisms (SNP) [29]. For instance, patients harboring CTLA-4 mutations that impair antitumor immunity are more likely to be responsive to anti-CTLA-4 immunotherapy. Moreover, CTLA-4 SNPs have been extensively studied in different cancers, including TNBCs [113]. Notably, specific SNPs in the promoter region of CTLA-4, such as CTLA-4c.49*G, are associated with breast cancer. Individuals with CTLA-4c.49*G alleles exhibit a 1.8-fold higher likelihood of developing breast cancer than those with the A/A genotype making anti-CTLA-4 a promising therapeutical approach for this patient group [12]. Treg cells are another example of potential biomarkers that could be used to elucidate a patient's responsiveness to anti-CTLA-4 therapy, since they modulate antitumor activity by affecting the Treg cell population [42]. Other potential prognostic and predictive biomarkers are TILs within the adjacent tumor stroma or focal areas [10, 123]. Prall et al. [81] reported that TILs and FOX3P + Treg immune cells were indicators of immunogenicity and prognosis in TNBC [81]. Furthermore, tremelimumab shows the highest responsiveness in invasive breast tumors with > 50% lymphocyte infiltration, also known as lymphocyte-predominant breast cancer (LPBC). Furthermore, a correlation exists between the number of tumor-infiltrating lymphocytes (TILs) and disease-free survival [105]. Stanton et al. [105] analyzed 256 TNBC tumors and demonstrated that with every 10% increase in TILs, there was a 17% reduction in the risk of recurrence (*p* = 0.023, HR 0.83, 95% CI 0.71–0.98) and a 27% decreased risk of death (*p* = 0.035, HR = 0.73; 95% CI 0.54–0.98) (Sherene [59]). Similarly, other reports have shown that high TILs levels after treatment are associated with good prognosis, as the greater the number of TILs, the more responsive the patients are to immunotherapy and chemotherapy [34, 64]. However, anti-CTLA-4 responsiveness prediction depends on the subtype and localization of TILs. For instance, patients with efficient CD8 + T-cell tumor infiltration had good treatment outcomes. In contrast, patients with accumulated CD8 + T cells in tumor-associated stroma had poor outcomes [1]. Recently, the WHO St. Gallen International Breast Cancer Conference allowed TILs quantification to determine the prognosis of patients with early-stage TNBC [9, 58].

These biomarkers shed light on the interplay between BC and the immune system, providing information on tumor responsiveness to treatment (Fig. 3). Technological advancements, such as single-cell omics, are promising as they would provide in-depth qualitative information on biomarkers with prognostic and predictive values for BC patients [54]. Overall, CTLA-4 SNP, TILs, and FOX3P + Treg cells are promising predictive biomarkers for anti-CTLA-4 therapy, but much more needs to be elucidated for anti-CTLA-4 personalized therapy in BC patients.

## Conclusion

Immunotherapy has revolutionized the treatment of several malignancies, and has only recently entered the treatment landscape of BC. However, the application of anti-CTLA-4 therapy presents challenges, including its limited effectiveness and considerable toxicity. Emerging immunotherapy data have shown that anti-CTLA-4 combination therapy could soon become the standard treatment for BC. Although very few anti-CTLA-4 clinical studies have been performed in patients with BC, this may change with a deeper understanding of CTLA-4 function in cancer immunity. Recent research highlighting anti-CTLA-4 combination strategies, such as Treg cell depletion, utilization of cancer vaccines, and consideration of the gut microbiome, has revealed promising preclinical results that are poised for subsequent investigation in patients. Future directions for anti-CTLA-4 therapy include second-generation antibodies and SMI with minimal irAE toxicities and high potency, and the identification of standard biomarkers for CTLA-4 immunotherapy. Finally, a comprehensive understanding of the role of anti-CTLA-4 mAB’s in cancer immunity is imperative for further immunotherapeutic advancements in patients with breast cancer.

## Data Availability

No datasets were generated or analysed during the current study.
